# Ethnic Identity in Transition: the Potential Impact of Ethnicity on Chronic Illness’ Medication Adherence in Post-Soviet Country

**DOI:** 10.1007/s40615-021-01048-x

**Published:** 2021-05-03

**Authors:** Kadi Lubi, Ain Raal, Pille Taba

**Affiliations:** 1 Health Education Center, Tallinn Health Care College, Kännu 67, 13418 Tallinn, Estonia; 2 Department of Health Technologies, Tallinn University of Technology, Akadeemia tee 15a, 19086 Tallinn, Estonia; 3 Institute of Pharmacy, Faculty of Medicine, University of Tartu, Nooruse 1, 50411 Tartu, Estonia; 4 Department of Neurology and Neurosurgery, Faculty of Medicine, University of Tartu, L. Puusepa 8, 50406 Tartu, Estonia; 5 Department of Neurology, Tartu University Hospital, L. Puusepa 8, 50406 Tartu, Estonia

**Keywords:** Information-seeking activity, Information sufficiency, Medication adherence, Chronic illness, Ethnicity

## Abstract

Previously, it has been shown that factors like ethnicity and proficiency of state’s official language not only influence self-management abilities and medication adherence but may also indicate the level of trust in physicians, medication, and healthcare system. This research aims to examine the potential impact of ethnicity on medication adherence based on the example of a post-Soviet country. The research was carried out as a quantitative survey among 303 hypertension and type 2 diabetes patients in Estonia, involving participants from ethnic majority and minority. Research was conducted in community pharmacies and data analysed statistically with SPSS. The findings were opposite to previous research. Although members of the ethnic minority used less illness-related sources, these sources relied more on evidence-based medicine compared to the ethnic majority. Because of this, medication adherence was also slightly higher for the ethnic minority compared to the majority. Therefore, these findings indicate trust in medical authorities, their decisions, and recommendations. There was a statistically significant relationship between general and illness-related information-seeking activity; however, medication adherence was not related to information-seeking activity. The research outlines that in addition to ethnolinguistic aspect, also potential cultural influence might determine the trust in medicine and medication adherence.

## Introduction

The occurrence of chronic diseases is increasing and taking the form of a silent pandemic [1]; more than half of all deaths have been caused by noncommunicable diseases [2], and it is estimated that, in developed countries, up to 57% of the population suffer from more than one chronic condition [3]. Medication nonadherence has been stated to be extremely common, which has remained an unmet challenge in managing chronic conditions [4, 5]. Previous research has shown that among other influencing factors, ethnic and racial minorities have lower levels of medication adherence [6–8] which might be due to not only proficiency of a state’s official language but also cultural practices [9]. Previous studies have highlighted different predictors for treatment adherence, e.g. the roles of treatment-related information, patient education, and counselling, pharmacists as educators [5], social support [10], motivated interviewing [4, 5], health literacy [11], and higher self-efficacy [12].

In their scoping review, Zhang et al. [9] have outlined that (among other factors) medication adherence is dependent on the proficiency of official language and cultural practices, which determine disparities in self-management and utilisation of health information and resources. Based on a literature review, it has been identified that African Americans as an ethnic minority do not always follow physicians’ instructions on medication administration compared to whites [6] and that they mistrust physicians, medications, and the healthcare system while tending to have belief in alternative medicine [13]. The study site was Estonia, a former Soviet country that is situated next to the Russian Federation and has gone through rapid developments (i.e. societal and (health) political developments) after the collapse of the Soviet Union (SU) [14]. The increase of ethnic Russians was related to World War II; and today, the Russian-speaking population constitutes almost one-third of the Estonian population [15]. As one of the consequences of occupation was also the requirement to learn and use the Russian language, it became a power attribute during the Soviet era [15]. Re-independence and transition of Estonia entailed polarisation of ethno-linguistic communities and challenge to Russian-speaking minority as they had to adjust to their new position in the context of the Estonian nation-state that changed Russians’ position in the society and required acquisition of Estonian as the new national language [15].

Health in SU was characterised by a paternalistic approach which precludes the role of the individual [16] and physicians were seen as gatekeepers to non-medical benefits [17]. The role of political ideology in former Soviet countries has been shown to influence the healthiness of the lifestyle, i.e. less healthy in countries where there is a desire for communism to return, which indicates the collective rather than individualistic health approach [18]. These aspects bring together multilevel problems in the context of health communication. First, Russian-speaking people might perceive language-derived barriers as due to historical roots there might not be willingness (or for younger generation also ability) among ethnic Estonians to speak Russian. Second, due to the traditional perception of physicians as gatekeepers, Russian-speaking people might not perceive the need for individual information-seeking as also according to a representative national survey Russian-speaking people are rather passive in terms of information-seeking [19]. An individual information search is highly evaluated, yet the content might not be fully trusted.

In the present analysis, we examine the potential impact of ethnicity on medication adherence based on the example of a post-Soviet country. We analyse information-seeking activity in terms of mass-media and illness-related information and its influence on medication adherence among members of the ethnic majority (Estonians) and the largest minority (Russians) in the context of hypertension (HTN) and type 2 diabetes (T2D).

## Methods

### Participants

In total, 303 participants were recruited in community pharmacies’ settings. The inclusion criteria were a diagnosis of HTN or T2D, adulthood (from age 18), the purchase of medications of HTN or T2D during the timeframe of data collection, and willingness to participate in a study. The exclusion criteria were age under 18 years and any kind of inability to fill out the questionnaire. The questionnaires were given a code, containing the number of the questionnaire, the indications of respondent’s gender (F/M), age, and illness (marked according to ATC diagnosis codes I10 and E10, respectively).

### Data Collection

The research was conducted as mixed-methods research by using a quantitative survey and qualitative in-depth semi-structured interviews from November 2018 to December 2019. The initial stage of the research was a quantitative survey and data were obtained from patients diagnosed with either HTN or T2D. Potential participants were approached with two questions to reveal whether they bought medication for themselves and whether they would like to participate in the study. After receiving oral consent, the aims of the research were introduced, and the written informed consent form was signed. The next step was answering the questionnaire, which was located on an electronic platform called eFormular.

The research was approved by the Human Research Ethics Committee of the University of Tartu (TBD later). The study is in line with the requirements of the Declaration of Helsinki and both EU and local data protection legislation.

### Measures

Information-seeking activity was assessed using a questionnaire that was shown to be feasible in a previous study on PD patients [20]. Respondents were provided with lists of sources (both for general and illness-related information). The participants assessed how much they used each of the sources to find either general or specific illness-related information. In order to measure medication adherence, questions about treatment adherence were added to the original questionnaire.

The questionnaire contained 36 questions. Out of these 36 questions, there were two that contained the list of 12 (for mass media) or 14 (for illness-related media) predefined information sources. The participants had to assess the usability of each source on a 4-point Likert scale (1, “never use the source”; 2, “rarely use the source”; 3, “sometimes use the source”; and 4, “mainly use the source” (for gathering information about general news or illness-related information)). One question contained twenty-one statements about respondents’ health and illness-related beliefs, experiences, or preferred modes of action. The participants had to assess these statements on a 5-point Likert scale on which 1 denoted “totally disagree”, 2 “partly disagree”, 3 “sometimes agree, sometimes disagree and so”, 4 “partly agree”, and 5 denoted “totally agree”. In addition, the questionnaire had sections about the socio-demographic background and illness-related information (e.g. general information about illness- and adherence-related behaviours, illness-related information availability and sufficiency).

### Analysis

The purpose of the statistical analysis was to analyse potential connections between information-seeking activity and medication adherence. Statistical analysis was performed with SPSS version 25 software, and two-tailed tests were used. The frequencies as well as means and standard deviations were calculated to describe sample characteristics. For cross-table analysis, Cramer’s V was used as a characteristic of the significance of the relationship between variables. A *p* value <0.05 was considered statistically significant. In order to examine information-seeking activity, two new aggregated index variables (based on previously described list of sources) and a recoding according to the number of used channels were used: variable “intensity and diversity of mass media usage” (low = 1–3; moderate = 4–6; high = 7–9; very high = 10–12) and “intensity and diversity of illness-related media usage” (low = 1–3; moderate = 4–7; high = 8–11; very high = 12–14). New compound variable for medication adherence was calculated based on whether the treatment regimen was followed and what the usual type of reaction was when the medication was missed (recoded as low, moderate, and high). To demonstrate the value of the information for the respondents, the correlation (by using Pearson’s correlation coefficients) between the variables of “how attainable is the information” and “how sufficient is the information” was analysed.

## Results

Table 1 summarises the characteristics of the study participants. Sixty per cent of the participants were women, which is in line with the findings of previous research [21, 22], and a majority (88.5%) were in the age range of 58–93 years (mean 71.1 ± 11.9). Half of the participants had graduated secondary school; more than one-third had any form of higher education. Although more than 70% of participants had had their illness(es) for more than 5 years, there were no participants who would have been members of any illness-related patient organisation. Sixty-three per cent of the participants defined themselves as Russians.

**Table 1:** Characteristics of the study participants

		No. of participants (*N*_total_ = 303)	Per cent
Gender	Male	121	39.9
	Female	182	60.1
Age	20–39	6	2
	39–58	43	14.2
	58–77	190	62.7
	77–94	64	21.1
Nationality	Estonian	112	37
	Russian or other	191	63
Education level	Basic	17	5.6
	Secondary school	166	54.8
	Applied higher/higher	120	39.6
More than 1 chronic illness	Comorbidity	125	41.3
	T2D	58	19.1
	HTN	120	39.6
Duration of illness, years	<5	79	26.1
	>5	224	73.9
No. of medicines used for the treatment	1 (including combination of 2 or more substances in 1)	98	32.3
	2–3	138	45.5
	4–5	41	13.5
	>5	26	8.6

Table 2 summarises the outcome of the bivariate correlation analysis of quantitative survey results regarding general and illness-related information-seeking activity and their relationship with medication adherence. The first column represents the correlation between general and illness-related information-seeking activity. Moderate to strong statistically significant correlations were found between these variables, indicating the role of previous information-seeking habits in illness-related information seeking. The analysis revealed that there were differences among moderate (40% Russians vs. 25% Estonians) or high (56% Russians vs. 71% Estonians) information-seeking activity groups (weak relationship (*r* = 0.151) but statistically significant (*p* = 0.03)). There was no ethnicity-based difference in very high information-seeking activity (4% for both ethnic groups).

**Table 2:** Correlations between general, illness-related information-seeking activity, and medication adherence

Chronic condition	General vs. illness-related info	Information-seeking activity for general info vs. medication adherence	Information-seeking activity for illness-related info vs. medication adherence
PD	0.221**	0.120	0.084
HTN	0.317**	0.081	0.072
T2D	0.382**	0.144	0.131
Total	0.275**	0.060	0.017

**Correlation is significant at the 0.01 level (2-tailed)

As seen from Table 2, there is no statistically significant relationship between information-seeking activity and medication adherence which is noteworthy as one would expect just the opposite. However, the findings reveal (Table 3) that while there is about a third of respondents in both groups with moderate adherence, there were more with higher medication adherence (63% vs. 59%) and less with low medication adherence (4% vs. 11%) among Russian-speaking respondents. Statistical relationship was weak (*r* = 0.140) but statistically significant (*p* ≤ 0.05).

**Table 3:** The amount of participants with different levels of medication adherence (aggregated variable, see sub-section “Analysis”)

	Low (*n*, %)	Moderate (*n*, %)	High (*n*, %)	Total, *n*
Estonians	12 (11%)	34 (30%)	66 (59%)	112
Russians	7 (4%)	63 (33%)	121 (63%)	191
Total, *n*	19	97	187	303

The usage of different sources and channels of illness-related information are summarised in Table 4. As seen from the table, regardless of their ethnicity, the most important information sources for chronically ill people are official evidence-based sources, e.g. both primary and speciality care physicians and materials distributed by them. While the importance of GPs was similar in both groups, the importance of specialists as an information source was remarkable. In addition, the difference in the usage of channels among ethnic groups with medium-sized intensity and statistical significance was revealed in the case of popular scientific health information from journals, TV, and radio. There were more Russian-speaking respondents who did not use these sources at all while a higher proportion of Estonian-speaking respondents tended to use these sources sometimes or even mainly. These findings indicate that the most trustworthy sources for illness-related questions are doctors for chronically ill people and ethnicity influences mainly the variety of other sources.

**Table 4:** The usage of different information sources based on identified ethnicity

	Not at all		Rarely		Sometimes		Mainly		Cramer V	*p* value
Source/ethnicity	Estonian	Russian	Estonian	Russian	Estonian	Russian	Estonian	Russian		
Health section in newspapers	29%	33%	22%	17%	31%	28%	18%	22%	0.083	0.555
Health magazines	34%	61%	18%	12%	28%	14%	20%	13%	0.267	0.000
Health sections of other magazines	44%	71%	22%	12%	20%	9%	14%	8%	0.268	0.000
Alternative health magazines	55%	79%	22%	11%	17%	6%	6%	4%	0.262	0.000
Health broadcasts in TV	23%	41%	15%	15%	35%	20%	27%	24%	0.201	0.006
Health broadcasts in radio	32%	62%	26%	17%	27%	12%	15%	9%	0.297	0.000
Illness-related webpages	54%	69%	13%	12%	15%	12%	18%	7%	0.183	0.018
Health-related webpages	55%	52%	15%	15%	20%	20%	10%	13%	0.044	0.901
Friends and acquaintances	35%	36%	24%	22%	33%	31%	8%	11%	0.0806	0.523
Family	20%	24%	24%	16%	35%	35%	21%	25%	0.112	0.282
GP	1%	1%	2%	2%	12%	12%	85%	85%	0.19	0.991
Specialist	6%	4%	9%	3%	14%	13%	71%	80%	0.148	0.86
Brochures/books from doctors	13%	14%	15%	9%	25%	21%	47%	56%	0.119	0.234
Patient organisation	88%	95%	3%	2%	5%	3%	4%	0%	0.163	0.045

Derived from the latter, information was considered highly accessible for 47% of participants. However, importantly, an equal number of participants (46%) said that while information is generally available, they still have some unanswered questions. For 6.3% of respondents, the available information is not sufficient. There was a strong, statistically significant relationship between information availability and sufficiency (Cramer’s V 0.427, *p* < 0.0001). Information availability and sufficiency had no significant association with the duration of illness and respondents’ education level. In more detail, it appears that people still miss basic information, as their unanswered questions vary from reasons for the illness to cures and treatment (including alternative options) and complication issues. The lack of explanations or reasoning can also be seen in cases in which people doubt their diagnosis or believe that the doctor is hiding something or not “honestly” telling the patient everything about the illness and related issues. Summarising these findings together with source-related findings, having direct medical communication to a main source may be a problem in the context of continuity of illness-related information. While there are structural barriers, e.g. limited accessibility to doctor’s appointments, situationally raised questions may remain unanswered which creates the perceptions of insufficiency in illness-related information. People with higher information-seeking activity may use other sources to try to find missing information; however, as the ability to understand and process achieved information may be limited, the overall result may be inappropriate actions as a patient or failure in self-management.

## Discussion

The results confirmed earlier findings of interconnections between general and illness-related information-seeking activity [20]. Although different medical sources were the most important ones regardless of the ethnicity, the analysis revealed that the variety of illness-related sources is slightly lower among members of ethnic minority, which may be related to a couple of aspects. First, lower proficiency of national language which has previously shown to influence self-management ability [9] and which in terms of individual information-seeking activity inhibits the access to available resources among traditional media channels. Second, cultural heritage of seeing physicians as gatekeepers [17], which might also contribute to lower desire or need to seek information individually.

The findings did not confirm the connection between higher information-seeking activity and better medication adherence as there was neither connection nor statistical significance found. Medication adherence is related to new practices accompanied with the illness and needs to be adapted to the existing lifestyle, the finding is supported by earlier research where it has been shown that the onset (i.e. diagnosis) of chronic illness does not entail new, illness-related, and illness-derived practices as people are keen on continuing existing lifestyle for as long as possible [23]. On the other hand, medication adherence (as an illness-related aspect) needs to be explained by healthcare professionals and (in addition to matching the existing lifestyle) the level of adoption is related also to the trust of healthcare professionals as authorities. Our findings indicated that despite the lack of relationship between information-seeking activity and medication adherence in general, medication adherence was slightly higher for Russian-speaking minority. The finding is controversial to previous findings, which have highlighted lower medication adherence for ethnic and racial minorities [7–9]. The reasons of our contradictory findings may be cultural and societal derived from the historical background that differs from the settings of previous researches. In the context of the present research, Russian-speaking ethnic minority has gone through several societal changes, including their own and Russian language’s position in the society [15]. Also, the authority of physicians, which has been culturally acquired [17], may have its influence expressed in the obedience to the instructions (i.e. medication adherence). However, as Cockerham et al. [18] have shown, the democratic approach to health issues might entail better outcomes. Thus, especially in keeping in mind the approaches of patient-centredness, individual responsibility, and unexpected public health emergencies (e.g. like COVID-19 pandemic), individual information-seeking should be continuously promoted and encouraged. The latter supports self-management, which is necessary in terms of chronic illnesses, but thereby it is also possible to mitigate the risk of information unavailability or insufficiency.

An important aspect to outline is the membership of patient organisation, which regardless of the ethnicity was rarely considered an illness-related information source. Previous research with PD patients has outlined that the importance of patient organisation increases together with the progression of the illness [20]. In the context of HTN and T2D, the nature of the illness might become important. While the symptoms of PD (e.g. moderate to severe motor symptoms) influence the management of daily activities, patients have practical needs to meet other patients and share their experience to gain knowledge about illness-related self-management [23] and this is something that HTN and T2D patients might never experience. The substantial difference in the nature of the illnesses might also have a negative impact on medication adherence and raising the hesitance whether medication is necessary at all or one should let the body to manage it.

More research is needed to examine whether cultural and ethnic influences appear also among the second and the third generations of ethnic minority.

## Limitations

Some limitations of the study should be acknowledged. The sample formation is based on the convenience sampling method due to which the study findings are generalisable neither to the wider society nor to all HTN and T2D diabetes sufferers. The convenience method itself is not a problem, and it is used in various occasions for the prominent research as well, but it is possible that with another type of sampling, the findings would be more precise. In the present case, the aspects of convenience influencing the overall results were that selected pharmacies were used for participant inclusion. Also, it is likely that more active people were involved leaving out people who are more passive in their information-seeking habits, which influences the results in terms of information-seeking activity. The aspect of activity might be relevant also in terms of gender division within the study sample—we had 60% of female participants who are considered to be more adherent to treatment regimen. In addition, a majority of participants were from a Russian-speaking minority, meaning they were overrepresented compared to their proportion of the general population in Estonia. However, as the aim was to examine the role of ethnicity, the authors believe that the opposite ratio in terms of ethnicity compared to the total structure of Estonian population is justified. Despite these limitations, the authors believe in the general value of new knowledge gained from the research and setting the directions for future research to investigate in the area of information-seeking behaviour and medication adherence in greater detail.

## Data Availability

Data will be made available on request.

## References

[1] Harris RE (2020). Epidemiology of chronic disease: Global perspectives.

[2] World Health Organization. Summary for Policymakers. Global status report on noncommunicable diseases. 2010:2011. 10.1017/CBO9781107415324.004.

[3] Hajat C, Stein E (2018). The global burden of multiple chronic conditions: a narrative review. Prev Med Rep.

[4] Kleinsinger F (2018). The unmet challenge of medication nonadherence. Perm J.

[5] Fernandez-Lazaro CI, García-González JM, Adams DP, Fernandez-Lazaro D, Mielgo-Ayuso J, Caballero-Garcia A, Moreno Racionero F, Córdova A, Miron-Canelo JA (2019). Adherence to treatment and related factors among patients with chronic conditions in primary care: a cross-sectional study. BMC Fam Pract.

[6] Gerber BS, Cho YI, Arozullah AM, Lee SYD (2010). Racial differences in medication adherence: a cross-sectional study of medicare enrollees. Am J Geriatr Pharmacother.

[7] Shaw SJ, Huebner C, Armin J, Orzech K, Vivian J (2009). The role of culture in health literacy and chronic disease screening and management. J Immigr Minor Health.

[8] Xie Z, Clair PS, Goldman DP, Joyce G (2019). Racial and ethnic disparities in medication adherence among privately insured patients in the United States. PLoS One.

[9] Zhang L, Gallagher R, Ding D, Neubeck L (2018). Self-management following a cardiac event in people of Chinese ethnicity living in western countries: a scoping review. J Immigr Minor Health.

[10] Lemstra M, Nwankwo C, Bird Y, Moraros J (2018). Primary nonadherence to chronic disease medications: a meta-analysis. Patient Prefer Adherence.

[11] Heijmans M, Waverijn G, Rademakers J, van der Vaart R, Rijken M (2015). Functional, communicative and critical health literacy of chronic disease patients and their importance for self-management. Patient Educ Couns.

[12] Al-Noumani H, Wu JR, Barksdale D, Sherwood G, AlKhasawneh E, Knafl G (2019). Health beliefs and medication adherence in patients with hypertension: a systematic review of quantitative studies. Patient Educ Couns.

[13] Hall GL, Heath M. Poor medication adherence in African Americans is a matter of trust. J Racial Ethn Health Disparities. 2020. 10.1007/s40615-020-00850-3. 10.1007/s40615-020-00850-3 33215358

[14] Habicht T, Reinap M, Kasekamp K, Sikkut R, Aaben L, Van Ginneken E (2018). Estonia: health system review. Health Systems in Transition.

[15] Vihalemm T, Kalmus V (2009). Cultural differentiation of the Russian minority. J Balt Stud.

[16] Field MG. The health and demographic crisis in post-Soviet Russia: a two-phase development. In: Field MG Twigg JL editors. Russ. torn Saf. nets. Heal. Soc. Welf. Dur. Transit. New York: St Martin’s Press 2000 11–42

[17] Haug MR (1976). The erosion of professional authority: a cross-cultural inquiry in the case of the physician. Milbank Mem Fund Q Health Soc.

[18] Cockerham WC, Hinote BP, Cockerham GB, Abbott P (2006). Health lifestyles and political ideology in Belarus, Russia, and Ukraine. Soc Sci Med.

[19] Vihalemm P, Kõuts-Klemm R. Meediakasutuse muutumine: internetiajastu saabumine [Changes in Media Use: Arrival in Internet Era]. In: Vihalemm P, Lauristin M, Kalmus V, Vihalemm T, editors. Eesti ühiskond kiirenevas ajas Uurin. “Mina. Maailm. Meedia” 2002–2014 tulemused [Estonian Soc. an Accel. time Find. Surv. “Me. World. Media” 2002–2014], Tartu: University of Tartu Press 2017 251–78.

[20] Lubi K, Vihalemm T, Taba P. Illness-related information seeking: the case of Parkinson’s disease patients. In: Lee G, editor. Adv. Educ. Res. 2nd ed., Delaware: Information Engineering Research Institute 2014 98–111.

[21] Villako P, Volmer D, Raal A (2012). Factors influencing purchase of and counselling about prescription and OTC medicines at community pharmacies in Tallinn, Estonia. Acta Pol Pharm Drug Res.

[22] Volmer D, Bell JS, Janno R, Raal A, Hamilton DD, Airaksinen MS (2009). Change in public satisfaction with community pharmacy services in Tartu, Estonia, between 1993 and 2005. Res Soc Adm Pharm.

[23] Lubi K (2019). The adaptation of everyday practices in the adoption of chronic illness. Heal An Interdiscip J Soc Study Heal.

